# Mesoporous SiO_2_ Nanoparticles: A Unique Platform Enabling Sensitive Detection of Rare Earth Ions with Smartphone Camera

**DOI:** 10.1007/s40820-018-0208-2

**Published:** 2018-06-01

**Authors:** Xinyan Dai, Kowsalya D. Rasamani, Feng Hu, Yugang Sun

**Affiliations:** Department of Chemistry, Temple University, 1901 North 13th Street, Philadelphia, PA 19122 USA

**Keywords:** Mesoporous silica nanoparticles, Rare earth ions, Quantitative detection, Antenna effect

## Abstract

Fast and sensitive detection of dilute rare earth species still represents a challenge for an on-site survey of new resources and evaluation of the economic value. In this work, a robust and low-cost protocol has been developed to analyze the concentration of rare earth ions using a smartphone camera. The success of this protocol relies on mesoporous silica nanoparticles (MSNs) with large-area negatively charged surfaces, on which the rare earth cations (e.g., Eu^3+^) are efficiently adsorbed through electrostatic attraction to enable a “concentrating effect”. The initial adsorption rate is as fast as 4025 mg (g min)^−1^, and the adsorption capacity of Eu^3+^ ions in the MSNs is as high as 4730 mg g^−1^ (equivalent to ~ 41.2 M) at 70 °C. The concentrated Eu^3+^ ions in the MSNs can form a complex with a light sensitizer of 1,10-phenanthroline to significantly enhance the characteristic red emission of Eu^3+^ ions due to an “antenna effect” that relies on the efficient energy transfer from the light sensitizer to the Eu^3+^ ions. The positive synergy of “concentrating effect” and “antenna effect” in the MSNs enables the analysis of rare earth ions in a wide dynamic range and with a detection limit down to ~ 80 nM even using a smartphone camera. Our results highlight the promise of the protocol in fieldwork for exploring valuable rare earth resources.

**Electronic supplementary material:**

The online version of this article (10.1007/s40820-018-0208-2) contains supplementary material, which is available to authorized users.

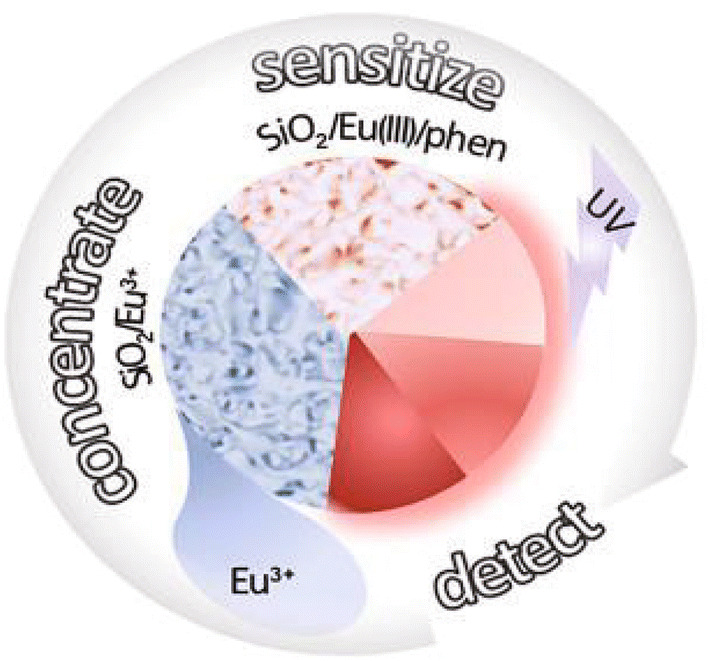

## Highlights

- A protocol for quantitative measurement of Eu^3+^ ions is developed using mesoporous silica nanoparticles.
- A “concentrating effect” of mesoporous silica nanoparticles is responsible for high adsorption capacity (4730 mg g^−1^) of Eu^3+^ ions. An “antenna effect” of 1,10-phenanthroline enables enhanced photoemission of adsorbed Eu^3+^ ions.
- The detection limit of Eu^3+^ ions is 80 nM even with smartphone camera.

## Introduction

The demand for rare earth metals has increased substantially due to their unique properties and applications in hi-tech products ranging from strong magnets, optical lenses, catalysts, aircraft engines, medical devices, and nuclear reactors [1–6]. However, the market supply is not sufficient because of the limited conservation, poorly developed extraction techniques, and export restrictions [7]. Therefore, exploring new resources of the valuable rare earth elements represents an urgent research direction [8–16]. The recent efforts include the search and extraction of rare earth metals from waste streams, coals, and industrial residues [8, 10, 16–19]. Sensitive detection of the low-concentration rare earth elements in these resources is crucial for precisely evaluating the economic value of various resources. The widely used methods include inductively coupled plasma mass spectrometry (ICP-MS), instrumental neutron activation analyses (INAA), X-ray fluorescence (XRF) and Raman spectroscopy. Despite the relatively good reproducibility and accuracy, these methods usually require tedious procedures, specialized detection environments, and expensive equipment. Developing low-cost and field-compatible protocols becomes essential to address the challenges.

In this report, we demonstrate a facile and cost-effective protocol for sensitive detection of rare earth ion with a very low detection limit down to 80 nM, in which Eu^3+^ ions have been used as a model. The success of this protocol relies on the use of mesoporous silica nanoparticles (MSNs) as a unique type of adsorbents, which can quickly adsorb Eu^3+^ ions via electrostatic attractions, to efficiently accumulate Eu^3+^ ions from very diluted solutions onto the MSNs. Due to the high density of mesoscale pores in the MSNs, the concentration of Eu^3+^ accumulated in the MSNs can be as high as tens of molars (mole L^−1^), which is denoted as “concentrating effect”. The optical transparency of SiO_2_ favors a simple approach to record intrinsic narrow-band fluorescent emissions of the rare earth elements. Some trivalent lanthanide ions, e.g., Eu^3+^ ions, usually have low photoexcitation efficiency because of a low light absorption cross-section and the forbidden transition of 4*f* orbitals [20, 21]. Forming complexes with sensitizing molecules that can strongly absorb light can transfer energy absorbed by the sensitizing molecules to the rare earth ions, leading to a strong fluorescent emission from the rare earth ions [22–24]. This sensitizing process refers to an “antenna effect”. For example, using 1,10-phenanthroline (phen) as sensitizing molecules can enhance the red fluorescent emission of Eu^3+^ ions under UV illumination. The 1,10-phenanthroline molecules efficiently absorb the UV irradiation and the excited molecules transfer energy to Eu^3+^ ions to promote their strong emission [25, 26]. Coordinating 1,10-phenanthroline molecules with Eu^3+^ adsorbed on the MSNs forms SiO_2_/Eu(III)/phen nanoparticles, which combines both the concentrating effect and the antenna effect to significantly increase the detection sensitivity of the red fluorescent emission of Eu^3+^. The intensified emission can be easily imaged with a smartphone camera to provide quantitative analysis.

## Experimental Methods

### Synthesis of Mesoporous Silica Nanoparticles

In a typical synthesis, 40 mg of poly(acrylic acid) (PAA, average M_W_ of 1800, Sigma Aldrich) was firstly dissolved in 1.5 mL of aqueous ammonia solution (28–30 wt%, Fisher Scientific). The resulting clear solution was added to 30 mL of ethanol (190 proof, Pharmco-Aaper) in a 100-mL round-bottom flask, while the solution was stirred at 500 revolutions per min (rpm) at room temperature. To this diluted PAA solution was added 750 μL of tetraethyl orthosilicate (TEOS, 98%, Sigma Aldrich) slowly. The TEOS liquid was added as five portions of 150 μL each with an interval of 2 h between two sequential additions to allow sufficient time for growing colloidal MSNs. After the last portion of TEOS was added, the reaction continued for 4 more h. The synthesized MSNs were collected after multiple cycles of centrifugation at 13,000 rpm and washing with deionized (DI) water. The obtained MSN powders were again washed with ethanol and dried in an oven held at 50 °C for 2 h.

### Concentrating Eu^3+^ on MSNs and Chemical Sensitization

In a typical concentrating process, 10 mL of ethanolic solution of EuCl_3_ (EuCl_3_·6H_2_O, 99.9% trace metal basis, Strem Chemicals, Inc.) with an appropriate concentration was added to 10 mL of ethanolic dispersion containing 2.5 mg of the as-synthesized MSNs. The resulting mixtures containing Eu^3+^ ions with concentrations of 1 mM, 100, 10, 1 μM, and 100 nM were stirred at 600 rpm at 70 °C for 30 min. Such incubation resulted in that all Eu^3+^ ions were adsorbed and concentrated in the MSNs, forming SiO_2_/Eu(III) colloidal particles. The SiO_2_/Eu(III) colloidal particles were then collected by a centrifugation at 13,000 rpm. To the SiO_2_/Eu(III) precipitated particles was added 20 mL ethanolic solution of 1 mM 1,10-phenanthroline (Sigma-Aldrich). Sonicating this mixture re-dispersed the SiO_2_/Eu(III) particles. The dispersion was then continuously stirred at 600 rpm for 30 min at 70 °C. The as-prepared SiO_2_/Eu(III)/phen nanoparticles were then centrifuged and washed multiple times with ethanol to remove excess phen molecules. The nanoparticles were dried in an oven maintained at 50 °C for 2 h and re-dispersed in 400 μL ethanol for colorimetric analysis. Reducing the dispersion volume from 20 mL to 400 μL resulted in a 50-time increase in the concentration of Eu^3+^ ions, representing a significant concentrating effect.

### Characterization

Absorption spectra of ethanolic solutions of EuCl_3_ with various concentrations were collected using a UV–Vis spectrophotometer (Thermo Scientific, Evolution 220). Fluorescence spectra were obtained using a fluorometer (PTI) with an analyzed software (PTI Felix32). Transmission electron microscopy (TEM) images of the MSNs were recorded with a JEOL TEM-1400 microscope operated at 120 kV. Energy-dispersive X-ray (EDX) analysis was carried out using a detector (X-Max^N^ 50, Oxford Instruments) equipped on a FEI Quanta 450 FEG scanning electron microscope operated at 30 kV. The digital images displaying the fluorescence of the SiO_2_/Eu(III)/phen nanoparticles were obtained under UV illumination (Kodak UVP Transilluminator TFM-20, excitation wavelength of 302 nm) using a smartphone camera. The images were processed using MATLAB software (R2017b) to analyze the average standard RGB intensity of pixels. Codes provided from MathWorks were used. The red intensities (corresponding to the fluorescence) of samples were then plotted against the concentration of Eu^3+^ ions of corresponding samples.

## Results and Discussion

**Fig. 1:**
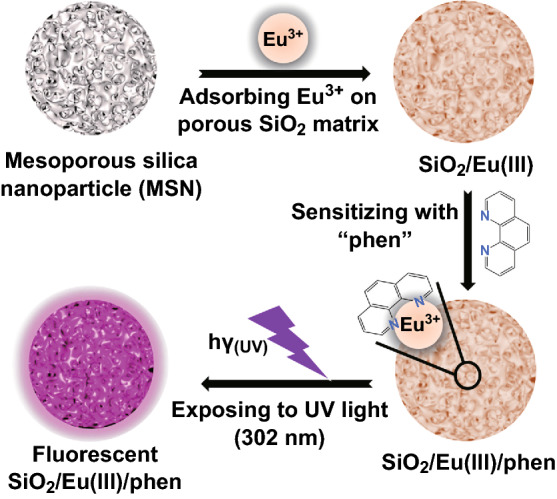
Schematic illustration of the major steps of the protocol for sensitive detection of Eu^3+^ ions from diluted solutions relying on the synergy of “concentration effect” and “antenna effect”

It is of great importance to develop a cost-effective and friendly strategy for detecting rare earth elements with superior sensitivity and low detection limits. For example, the colorimetric detection of Eu^3+^ ions in liquid solutions using traditional UV–Vis absorption and fluorescence spectroscopy provides the detection limits of only ~ 2 and ~ 0.1 mM, respectively (Fig. S1). These values are far from satisfactory to be applied for an on-site survey of low-concentration samples. To enable the colorimetric analysis of the low-concentration rare earth elements, one straightforward strategy is to concentrate the dilute species into a much smaller volume through efficient adsorption on porous solids. MSNs with super high surface areas represent a promising class of adsorbents because the optical transparency of silica is compatible with colorimetric analysis. As depicted in Fig. 1, the MSNs are firstly synthesized via a sol–gel process in the presence of PAA colloids that serve as soft templates to promote the growth of silica nanoparticles [27]. Selective dissolution of the PAA results in mesoscale pores in the silica nanoparticles. The MSNs exhibit negative surface charges, originated from the abundant –Si–O^−^ groups, which can efficiently adsorb positively charged Eu^3+^ ions through electrostatic attraction (Fig. 1). The high-density pores in the MSNs provide high adsorption capacity toward Eu^3+^, thus strong “concentrating effect”.

The intrinsic weak optical absorption coefficient of Eu^3+^ causes the low detection sensitivity of traditional UV–Vis absorption and fluorescence spectroscopies (Fig. S1). To overcome this limitation, the concentrated Eu^3+^ ions can be sensitized with molecules that can strongly absorb light and transfer energy to the Eu^3+^ ions. For example, soaking the MSNs with adsorbed Eu^3+^ ions, which are labeled as SiO_2_/Eu(III) nanoparticles for convenience, in a solution containing the sensitizer of 1,10-phenanthroline (phen), forming phen-Eu^3+^ complex in the silica matrix (step ii, Fig. 1). The *π*–*π* transition in 1,10-phenanthroline molecules makes them strongly absorb UV light [23, 28]. The photon energy absorbed in 1,10-phenanthroline can then efficiently transfer to Eu^3+^ ions in the phen-Eu^3+^ complex to excite the Eu^3+^ ions [21], resulting in a red fluorescent emission much stronger than the directly excited Eu^3+^ ions (Fig. 1). The enhancement in fluorescence of Eu^3+^ ions originates from the efficient energy transfer from the sensitizer molecules, which behave like antennas to strongly absorb light. This mechanism is called an “antenna effect”. The emitted red light can be directly observed without assistance of instrumentation even for the samples with very low concentration of Eu^3+^ ions. The synergy of “concentrating effect” and “antenna effect” in the MSNs enables the promise in developing a convenient, low-cost, highly sensitive protocol for analyzing rare earth elements.

**Fig. 2:**
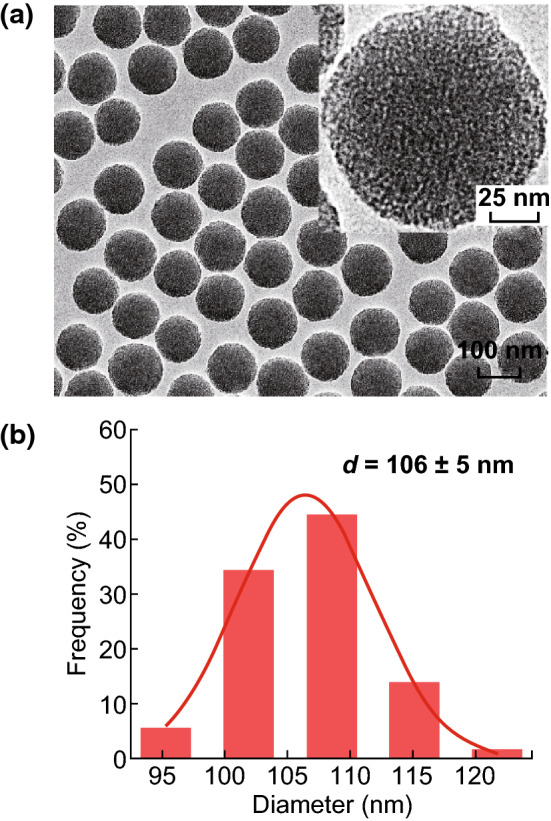
**a** TEM image and **b** size distribution histogram of the synthesized mesoporous silica nanoparticles (MSNs)

Figure 2a presents a typical TEM image of the synthesized MSNs, which are highly uniform in size with an average diameter of 106 nm (Fig. 2b) and spherical geometry. The magnified TEM image (inset in Fig. 2a) of individual MSN reveals the three-dimensional (3D) hierarchical porous structure in each MSN. In addition, the MSNs exhibit highly negatively charged surfaces with a zeta potential of − 122 ± 25 mV. The yield of MSNs synthesized via the PAA-assisted sol–gel process [25] is close to 100%, which has been verified by weighing the mass of the dried product followed by comparing with the value calculated from the reaction stoichiometry. The high yield and feasibility of large-scale synthesis are critical for developing cost-effective applications using the MSNs shown in Fig. 2.

The high porosity and high-density negative charges in the MSNs are favorable for efficiently adsorbing Eu^3+^ cations via electrostatic attraction. Three parameters including adsorption kinetics, the time required to reach adsorption equilibrium, and adsorption capacity, are taken into account to assess the adsorption ability of the MSNs. The adsorption of Eu^3+^ can reach the equilibrium after the EuCl_3_ solution is mixed with the MSNs for ~ 15 min at 70 °C. The adsorption capacity is as high as ~ 4730 mg g^−1^ (Eu^3+^/MSNs) (Fig. S2). Data fitting reveals that the adsorption of Eu^3+^ on the MSNs follows a pseudo-second-order kinetics with the initial adsorption rate of 4025 mg (g min)^−1^ (Fig. S2). The superior adsorption kinetics and adsorption capacity make the MSNs a promising platform for quickly concentrating trace lanthanide ions from dilute solutions.

**Fig. 3:**
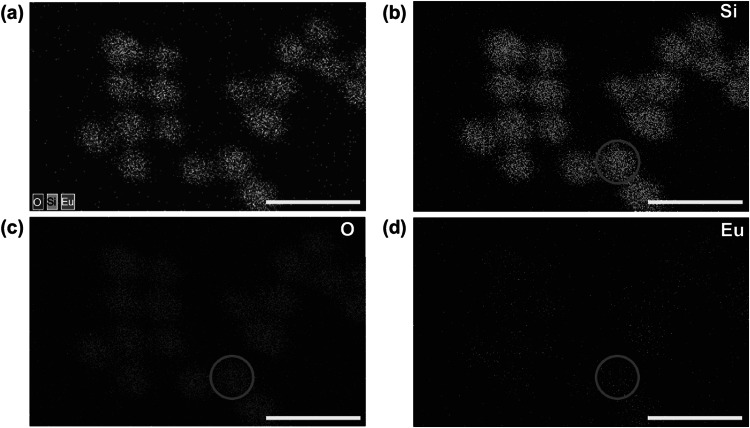
EDX elemental mapping images of **a** overlaid Si, O, and Eu elements, **b** Si, **c** O, **d** Eu for SiO_2_/Eu(III)/phen nanoparticles. The scale bars represent 250 nm. The red circles in (**b**–**d**) highlight the same nanoparticle. (Color figure online)

EDX analysis has been conducted to verify the uptake of Eu^3+^ ions in the MSNs. Figure 3a presents EDX mapping of Si, O, and Eu over an assembly of SiO_2_/Eu(III) nanoparticles. The observed EDX signal of Eu indicates that Eu^3+^ ions are indeed adsorbed to the MSNs (Fig. 3c). The perfect overlap of the EDX images of all three elements further confirms that all the MSNs are capable of adsorbing Eu^3+^ ions (Fig. 3d). The spatial distribution of Eu signal in an individual nanoparticle (highlighted by the red circles) is relatively uniform, implying that Eu^3+^ ions diffuse into the interconnected mesoscale pores in the MSNs in addition to the adsorption on the outer nanoparticle surfaces. The MSNs with adsorbed Eu^3+^ (i.e., SiO_2_/Eu(III) nanoparticles) are easily collected via centrifugation to complete the concentrating process. Assuming the porosity decreases the mass density of the MSNs to 50% of the value of SiO_2_ (2.65 g cm^−3^), the MSNs with a volume of 1 cm^3^ can adsorb Eu^3+^ ions up to 6.267 g given the maximum adsorption capacity of 4730 mg g^−1^. This value corresponds to a concentration of 41.2 M that is much larger than the Eu^3+^ concentrations in sample solutions (usually on the level of μM or nM).

Soaking the SiO_2_/Eu(III) nanoparticles in a solution of 1,10-phenanthroline molecules can form a complex with the adsorbed Eu^3+^ ions. Forming the phen-Eu^3+^ coordination bonds enables the efficient energy transfer from the 1,10-phenanthroline molecules capable of strongly absorbing UV light, to the Eu^3+^ ions, which exhibit low optical absorption cross-section and forbidden *f*–*f* transitions [20]. Such an energy transfer results in the strong emission of Eu^3+^ under UV illumination, facilitating a sensitive detection of Eu^3+^ ions by probing the red fluorescent emission (Fig. S3, top). It is worthy of note that the lack of optical absorption of visible light in SiO_2_ favors the efficient extraction of the red emission from the SiO_2_/Eu(III)/phen nanoparticles to the detector. The intensity of fluorescence varies with the amount of the SiO_2_/Eu(III)/phen nanoparticles assembled on a supporting substrate. Reliable and quantitative analysis requires the precise control over the thickness and uniformity of nanoparticle films, which is nontrivial and very difficult to achieve, in particular for fieldwork.

**Fig. 4:**
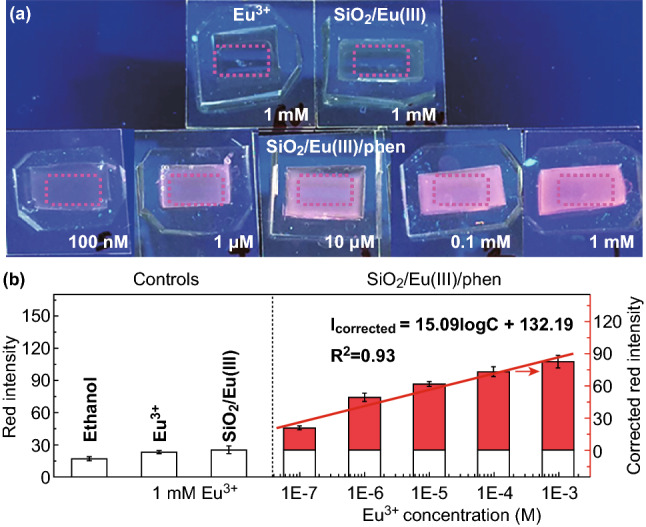
**a** A digital photograph of various solution samples under an UV lamp with a wavelength of 302 nm. Each sample represents 400 uL ethanolic solution (or dispersion) of the corresponding nanoparticles confined in PDMS pockets. The samples in the top row are 1 mM EuCl_3_ solution and SiO_2_/Eu(III) nanoparticle dispersion with Eu^3+^ ions adsorbed from 1 mM EuCl_3_ solution. The bottom row shows the SiO_2_/Eu(III)/phen nanoparticle dispersions Eu^3+^ ions adsorbed from EuCl_3_ solutions with concentrations from 100 nM to 1 mM (from left to right). **b** Quantized red emission intensities of various samples shown in **a**. (Color figure online)

To avoid the preparation of uniform films of SiO_2_/Eu(III)/phen nanoparticles, we can disperse a known amount of the nanoparticles in a known volume of solvent (e.g., ethanol) to make a high-centration dispersion, which is convenient to achieve precisely and repeatedly. Despite the dispersion of SiO_2_/Eu(III)/phen nanoparticles in the solvent decreases the concentration of Eu^3+^, the concentration of Eu^3+^ can be still much higher than the source solution when the volume of solvent used to re-disperse the SiO_2_/Eu(III)/phen nanoparticles is small enough. For example, adsorbing Eu^3+^ ions from 20 mL solution of 1 μM EuCl_3_ to 2.5 mg MSNs followed by re-dispersing the corresponding SiO_2_/Eu(III)/phen nanoparticles in 0.4 mL ethanol, resulting in 50 times concentration. The red emission from the SiO_2_/Eu(III)/phen nanoparticle dispersion can be imaged directly with a smartphone camera by placing the dispersion in a transparent polydimethylsiloxane (PDMS) mold with a rectangular pocket (~ 0.8 × 1.8 cm^2^) laminated on a glass slide (Fig. 4a). In contrast, no red emission is detected for the solution containing 1 mM Eu^3+^ and the dispersion of SiO_2_/Eu(III) nanoparticles without sensitization of 1,10-phenanthroline. The comparison highlights that the synergy of “concentrating effect” and “antenna effect” dramatically enhances the detection sensitivity of Eu^3+^ ions by using the MSNs as a platform. Figure 4a presents the digital images of the SiO_2_/Eu(III)/phen nanoparticle dispersions prepared from stock solutions with different Eu^3+^ concentrations, i.e., 100 nM, 1, 10 μM, 0.1, and 1 mM (from left to right). The photographs show the red emission intensity increases with the concentration of Eu^3+^ under UV illumination. The emission intensity recorded in the digital photographs can be quantitatively analyzed. Extracting the RGB information of total 5000 pixels (100 pixels in width and 50 pixels in height) highlighted with the dashed rectangles (Fig. 4a) allows an estimation of the averaged red intensities (details available in Supporting Information).

A solvent of ethanol (190 proof), a solution of 1 mM EuCl_3_, and a dispersion of the SiO_2_/Eu(III) nanoparticles exhibit red intensities of 16–24 that mainly originate from weak fluorescence of ethanol–water clusters [26] and ethanolic EuCl_3_ solution [29] (Fig. 4b). With the formation of phen-Eu^3+^ complex, the red intensities of the dispersion of SiO_2_/Eu(III)/phen nanoparticles increase significantly, exhibiting the values from 44 to 106 for the samples prepared from the Eu^3+^ solutions with concentrations from 100 nM to 1 mM (Fig. 4b). The actual intensity of emission from the phen-Eu^3+^ complex in MSNs is calculated with a subtraction of the maximum intensity of the control samples (i.e., 24), and the corrected intensity, *I*_corrected_, of a sample is presented in Fig. 4b (solid red post). The corrected red intensity exhibits a linear-logarithmic dependence on the concentrations (*C*, in unit M) of Eu^3+^ ions in the source solutions, i.e., *I*_corrected_ = 15.09 × log*C* + 132.19 (Fig. 4b). The corresponding detection limit is 82.4 nM according to three times of the maximum deviation of the measurement (3*σ*_max_ = 17.6). The detection limit can be further improved by increasing the volume of source Eu^3+^ solutions to concentrate more Eu^3+^ in the dispersion of the SiO_2_/Eu(III)/phen nanoparticles. The dynamic detection range is broad in a range of tens of nM to tens of mM.

## Conclusion

The combination of concentrating effect, which corresponds to adsorption of Eu^3+^ to the large-area surfaces of the MSNs, and antenna effect, which originates from the energy transfer of sensitizing 1,10-phenanthroline molecules with strong UV absorption to Eu^3+^ ions, for the first time, enables the quick detection of rare earth elements without the sophisticated instrumentation. The use of low-cost MSNs as the platform of detection is critical because (1) the high-density mesoscale pores in the MSNs and high-density negative surface charges enable a quick adsorption of large amount of rare earth ions through electrostatic attraction; (2) the large size (e.g., ~ 100 nm) of the MSNs favors the easy collection of the MSNs with adsorbed rare earth ions even through simple centrifugation; and (3) the optical transparency and large refractive index of SiO_2_ benefit the efficient extraction of light emission from the rare earth ions adsorbed in the pores of the MSNs when optical fluorescence spectroscopy is used for analysis. This protocol is promising for applicable field detection of the strategic rare earth resources, usually with very low concentrations.

The presence of rare earth elements can be directly visualized with eyes or smartphone cameras from the films/dispersions of MSNs with adsorbed rare earth ions and sensitizing molecules. Quantitative analysis can be achieved by processing the digital photographs with MATLAB to determine the concentration of samples according to the calibration curve. The accuracy and sensitivity of this protocol can be further improved by using color-specific cameras, more efficient sensitizing molecules, and MSNs with larger surface areas. Due to the specialty of sensitizing molecules, it is necessary to design and synthesize efficient sensitizers to apply this protocol to sensing various rare earth element ions. Although the fluorescence of Eu^3+^ is barely influenced by the presence of alkali metal ions (e.g., Na^+^) and alkaline metal ions (e.g., Ca^2+^), the fluorescence of Eu^3+^ detected by the smartphone camera might be significantly influence by some coexisting transition metal ions (e.g., Fe^2+^ and Cu^2+^), which induce charge transfer from Eu^3+^ to them [30, 31]. Therefore, if there is a field sample containing the interfering transition metal ions, an appropriate pretreatment such as ion exchange, solvent extraction, and electromembrane separation [32–35] is necessary to selectively remove the major coexisting transition metal ions, eliminating their potential influence on the detection of rare earth metal ions.

## Electronic supplementary material

Below is the link to the electronic supplementary material.

Supplementary material 1 (PDF 464 kb)

## References

[1] Gai S, Li C, Yang P, Lin J (2013). Recent progress in rare earth micro/nanocrystals: soft chemical synthesis, luminescent properties, and biomedical applications. Chem. Rev..

[2] Goodenough KM, Wall F, Merriman D (2017). The rare earth elements: demand, global resources, and challenges for resourcing future generations. Nat. Resour. Res..

[3] Boonsin R, Chadeyron G, Roblin JP, Boyer D, Mahiou R (2015). Development of rare-earth-free phosphors for eco-energy lighting based LEDs. J. Mater. Chem. C.

[4] Haranath D, Mishra S, Joshi AG, Sahai S, Shanker V (2011). Effective doping of rare-earth ions in silica gel: a novel approach to design active electronic devices. Nano-Micro Lett..

[5] John R, Rajakumari R (2012). Synthesis and characterization of rare earth ion doped nano ZnO. Nano-Micro Lett..

[6] Pu Y, Tang K, Zhu DC, Han T, Zhao C, Peng LL (2013). Synthesis and luminescence properties of (Y, Gd) (P, V)O_4_:Eu^3+^, Bi^3+^ red nano-phosphors with enhanced photoluminescence by Bi^3+^, Gd^3+^ doping. Nano-Micro Lett..

[7] Massari S, Ruberti M (2013). Rare earth elements as critical raw materials: focus on international markets and future strategies. Resour. Policy.

[8] Lin R, Howard BH, Roth EA, Bank TL, Granite EJ, Soong Y (2017). Enrichment of rare earth elements from coal and coal by-products by physical separations. Fuel.

[9] Roth E, Bank T, Howard B, Granite E (2017). Rare earth elements in Alberta oil sand process streams. Energy Fuels.

[10] Wilfong WC, Kail BW, Bank TL, Howard BH, Gray ML (2017). Recovering rare earth elements from aqueous solution with porous amine–epoxy networks. ACS Appl. Mater. Interfaces.

[11] Dutta T, Kim KH, Uchimiya M, Kwon EE, Jeon BH, Deep A, Yun ST (2016). Global demand for rare earth resources and strategies for green mining. Environ. Res..

[12] Maes S, Zhuang WQ, Rabaey K, Alvarez-Cohen L, Hennebel T (2017). Concomitant leaching and electrochemical extraction of rare earth elements from Monazite. Environ. Sci. Technol..

[13] Noack CW, Dzombak DA, Karamalidis AK (2015). Determination of rare earth elements in hypersaline solutions using low-volume, liquid–liquid extraction. Environ. Sci. Technol..

[14] Roosen J, Van Roosendael S, Borra CR, Van Gerven T, Mullens S, Binnemans K (2016). Recovery of scandium from leachates of Greek bauxite residue by adsorption on functionalized chitosan–silica hybrid materials. Green Chem..

[15] Kim D, Powell LE, Delmau LH, Peterson ES, Herchenroeder J, Bhave RR (2015). Selective extraction of rare earth elements from permanent magnet scraps with membrane solvent extraction. Environ. Sci. Technol..

[16] Funari V, Bokhari SNH, Vigliotti L, Meisel T, Braga R (2016). The rare earth elements in municipal solid waste incinerators ash and promising tools for their prospecting. J. Hazard. Mater..

[17] D. Golightly, F.O. Simon, *Methods for sampling and inorganic analysis of coal* (US Government Printing Office, 1989), pp. 35–57

[18] Pinto FG, Junior RE, Saint’Pierre TD (2012). Sample preparation for determination of rare earth elements in geological samples by ICP-MS: a critical review. Anal. Lett..

[19] T.L. Bank, E.A. Roth, P. Tinker, E. Granite, *Analysis of rare earth elements in geologic samples using inductively coupled plasma mass spectrometry*, US DOE Topical Report-DOE/NETL-2016/1794 (No. NETL-PUB-20441). National Energy Technology Lab (NETL), Pittsburgh, PA, 2016. 10.2172/1415779

[20] Rocha LA, Freiria J, Caiut JM, Ribeiro SJL, Messaddeq Y, Verelst M, Dexpert-Ghys J (2015). Luminescence properties of Eu-complex formations into ordered mesoporous silica particles obtained by the spray pyrolysis process. Nanotechnology.

[21] Ishii A, Hasegawa M (2015). An interfacial europium complex on SiO_2_ nanoparticles: reduction-induced blue emission system. Sci. Rep..

[22] Taydakov IV, Akkuzina AA, Avetisov RI, Khomyakov AV, Saifutyarov RR, Avetissov IC (2016). Effective electroluminescent materials for OLED applications based on lanthanide 1.3-diketonates bearing pyrazole moiety. J. Lumin..

[23] Ishii A, Hasegawa M (2016). The ethanol-induced interfacial reduction of a europium complex on SiO_2_ nanoparticles. Chem. Lett..

[24] Jiang L, Zheng JW, Chen WC, Ye JJ, Mo LE (2017). Tuning coordination environment: better photophysical performance of europium (iii) complex. J. Phys. Chem. C.

[25] Wan Y, Yu SH (2008). Polyelectrolyte controlled large-scale synthesis of hollow silica spheres with tunable sizes and wall thicknesses. J. Phys. Chem. C.

[26] Liu Y, Song CY, Luo XS, Lu J, Ni XW (2007). Fluorescence spectrum characteristic of ethanol–water excimer and mechanism of resonance energy transfer. Chin. Phys..

[27] Wan Y, Zhao DY (2007). On the controllable soft-templating approach to mesoporous silicates. Chem. Rev..

[28] Tosonian S, Ruiz CJ, Rios A, Frias E, Eichler JF (2013). Synthesis, characterization, and stability of iron (III) complex ions possessing phenanthroline-based ligands. Open J. Inorg. Chem..

[29] Lochhead MJ, Wamsley PR, Bray KL (1994). Luminescence spectroscopy of europium(III) nitrate, chloride, and perchlorate in mixed ethanol–water solutions. Inorg. Chem..

[30] Bünzli JC, Piguet C (2005). Taking advantage of luminescent lanthanide ions. Chem. Soc. Rev..

[31] Nonat AM, Harte AJ, Senechal-David K, Leonard JP, Gunnlaugsson T (2009). Luminescent sensing and formation of mixed f–d metal ion complexes between a Eu(III)-cyclen-phen conjugate and Cu(II), Fe(II), and Co(II) in buffered aqueous solution. Dalton Trans..

[32] Spedding FH, Powell JE, Wheelwright EJ (1994). The separation of adjacent rare earths with ethylenediamine-tetraacetic acid by elution from an ion-exchange resin. J. Am. Chem. Soc..

[33] Xie F, Zhang TA, Dreisinger D, Doyle F (2014). A critical review on solvent extraction of rare earths from aqueous solutions. Miner. Eng..

[34] Martoyan GA, Karamyan GG, Vardan GA (2016). New technology of extracting the amount of rare earth metals from the red mud. IOP Conf. Ser.: Mater. Sci. Eng..

[35] Hoogerstraete TV, Wellens S, Verachtert K, Binnemans K (2013). Removal of transition metals from rare earths by solvent extraction with an undiluted phosphonium ionic liquid: separations relevant to rare-earth magnet recycling. Green Chem..

