# Supplementary material for: Mesoporous SiO2 Nanoparticles: A Unique Platform Enabling Sensitive Detection of Rare Earth Ions with Smartphone Camera
Source: Nanomicro Lett. 2018 Jun 1;10(4):55. doi: 10.1007/s40820-018-0208-2 (PMC6199100; doi:10.1007/s40820-018-0208-2)
Supplement: Supplementary file 1 — Supplementary material 1 (PDF 464 kb) [file 40820_2018_208_MOESM1_ESM.pdf]

Supporting Information for

Mesoporous SiO_2 Nanoparticles: A Unique Platform Enabling Sensitive Detection of Rare Earth Ions with Smartphone Camera

Xinyan Dai[1, †], Kowsalya D. Rasamani[1, †], Feng Hu[1], Yugang Sun[1,*]

[1]Department of Chemistry, Temple University, 1901 North 13th Street, Philadelphia, Pennsylvania 19122, USA

†These authors contributed equally.

*Corresponding author. E-mail: ygsun@temple.edu (Yugang Sun)

Phone: 215-204-4327, Fax: (215) 204-1532

S1 Conventional Spectroscopy Analysis

The UV-Vis absorption and fluorescence spectroscopy were performed to evaluate the sensitivity for detecting Eu^{3+} ions. The UV-Vis absorption spectra of ethanolic solutions of $EuCl_3$ with varying concentrations are presented Fig. S1a. The broad absorption peak centered at 396 nm corresponds to the F-L transition of Eu (i.e., $^7F_0$-$^5L_6$ energy states). According to Beer law, the peak intensity linearly depends on the concentration of Eu^{3+} ions, showing a detection limit of ~2 mM (Fig. S1b).

The fluorescence spectra of the dimethylformamide (DMF) solution of $EuCl_3$ exhibit a number of emission peaks at 578, 591, 612, 649, and 694 nm, which correspond to the energy transitions of $^5D_0$-$^7F_0$, $^5D_0$-$^7F_1$, $^5D_0$-$^7F_2$, $^5D_0$-$^7F_3$, and $^5D_0$-$^7F_4$, respectively (Fig. S1c) [1]. The intensity of the peak at 612 nm (red in color) is linearly dependent on the concentration of Eu^{3+} ions, showing a detection limit of ~0.1 mM (Fig. S1d).

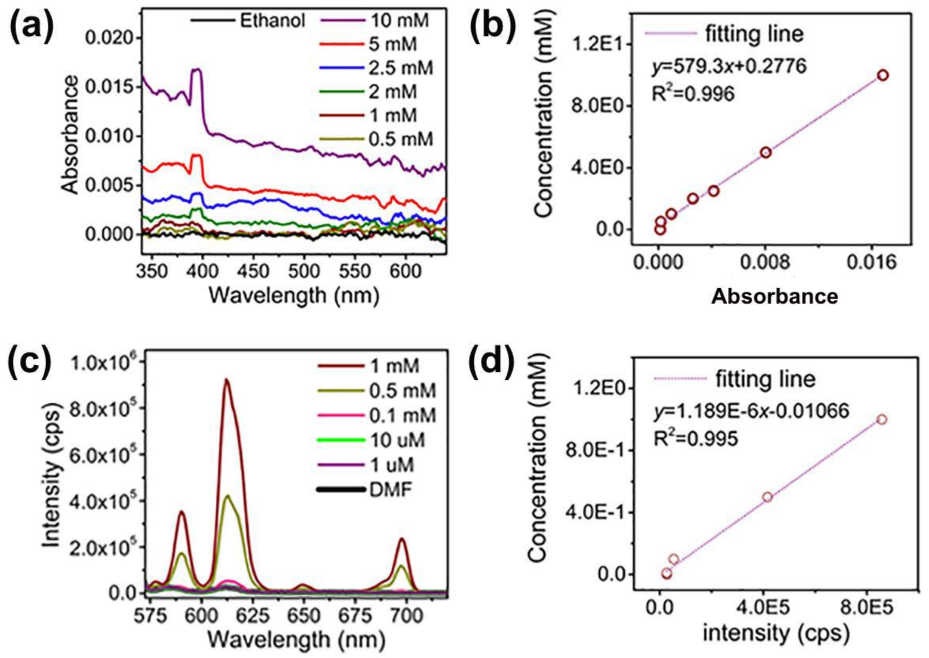

Fig. S1 **a** UV-Vis absorption spectra of ethanolic solutions of $EuCl_3$ with different concetrations and **b** the relationship of the peak intensity at 396 nm and the concentration of Eu^{3+} ions. **c** Fluorescence spectra of $EuCl_3$ solutions in DMF recorded with an excitation wavelength of 280 nm and **d** the relationship of the peak intensity at 612 nm and the concentration of $EuCl_3$.

S2 Adsorption Kinetics of Eu^{3+} on MSNs

The kinetics of adsorbing Eu^{3+} ions on the MSNs were evaluated by continuously monitoring the decrease of concentration of Eu^{3+} ions after the Eu^{3+} ions were mixed the MSNs. In a typical study, 2.5 mg of the MSN powder was added to 20 mL ethanolic solution of 5 mM $EuCl_3$ that was maintained at 70 °C. At different incubation times (i.e., 0, 5, 15, 25, and 45 min), aliquots of 3 mL solution were sampled, cooled down to room temperature, and centrifuged at 13.4 K rpm for 30 min. The supernatants were analyzed with UV-Vis spectroscopy to determine the concentrations of Eu^{3+} ions (Fig. S2a), and the amount of Eu^{3+} ions adsorbed to the MSNs were calculated accordingly. The specific adsorption at time (t), Q_t, was calculated as the ratio of the mass of the adsorbed metal ions to the total mass of the MSNs. The calculation used the initial concentration of Eu^{3+} ions (C_0, in unit mM), the concentration of Eu^{3+} ions in the supernatants at t (C_t, in unit mM), the initial volume of the solution (V, in unit L), the molar mass of Eu (M_{Eu}, in unit g/molar), and the mass of the MSNs (m_{MSN}, in unit g). The specific adsorption, Q_t (in unit mg g^{-1}), was calculated by

$$Q_t = \frac{(C_0 - C_t) \cdot V \cdot M_{EU}}{m_{MSN}}$$

The time evolution of of Q_t is presented in Fig. S2b. The results were analyzed using the quasi 1st order and 2nd order models [2].

1st order: $Q_t = Q_e (1- \exp(k_1 t))$

2nd order: $Q_t = \frac{k_2 Q_e^2 t}{1+k_2 Q_e t}$

k_1 (min^{-1}) and k_2 (g (mg min)$^{-1}$) refer to the rate constants, and Q_e (mg g^{-1}) refers to maximum adsorption capacity at the adsorption equilibrium. The fitting shows the equations Q_t=4729(1-exp(-0.166t))for the quasi 1st order kinetics and Q_t=4025x/(1+0.85x) for the quasi 2nd order kinetics, indicating the adsorption of Eu^{3+} ions on the MSNs obeys more like the quasi 2nd order kinetics. The initial adsorption rate is $k_2 Q_e^2 = 4025$ mg (g min)$^{-1}$ and maximum adsorption capacity Q_e=4025/0.85=4730 mg g^{-1}.

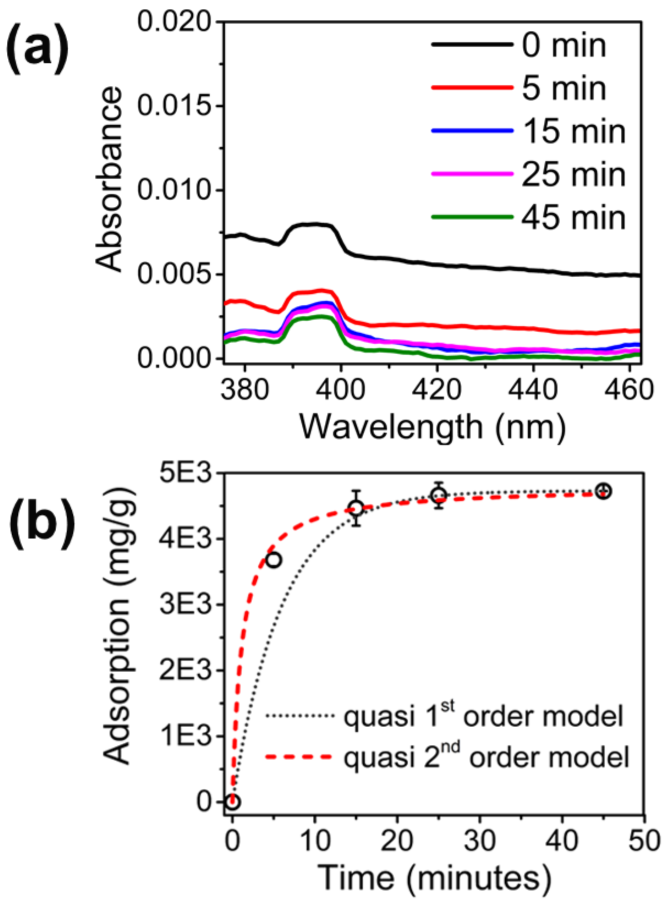

Fig. S2 a UV-Vis absorption spectra of the supernatants sampled at different incubation times. **b** Calculated specific adsorption of Eu^{3+} ions (in mg) on the MSNs (in g) as a function of the incubation time. The dashed curves represent the fitting according to the quasi 1st order and 2nd order kinetic models.

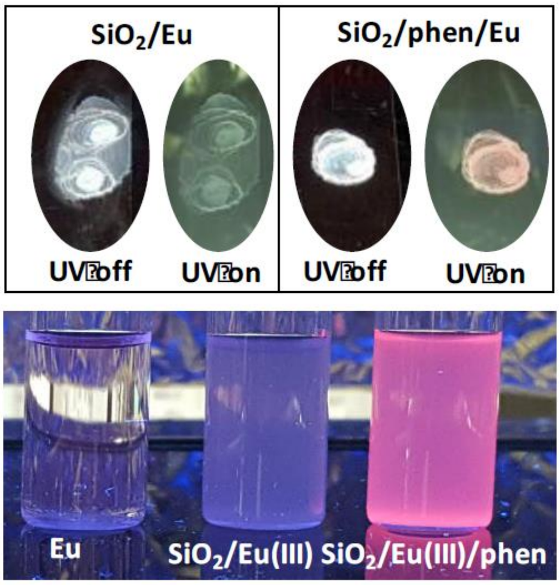

Fig. S3 (Top) Digital photos of dry films of the SiO_2/Eu(III) nanoparticles and the SiO_2/Eu(III)/phen nanoparticles in the absence and presence of UV irradiation. The nanoparticles adsorbed Eu^{3+} ions from 20 mL of 5 mM $EuCl_3$ solution. (Bottom) Digital photo of the solution/dispersions containing 25 mM Eu^{3+}, SiO_2/Eu(III) with a concentration of 5 mM Eu^{3+}, and SiO_2/Eu(III)/phen with a concentration of 5 mM Eu^{3+} under UV irradiation.

S4 Analysis of Digital Photos

The digital photos of the red emission were analyzed using Matlab. A randomly selected area of 100 pixels ×50 pixels for each sample was cropped with Photoshop software. The cropped image of each sample was imported into Matlab software. The code for averaged RGB information for each pixel is available in the online manual at Mathwork website. At least three measurements for different selected areas for each sample were repeated. It is noted that the brightness and contrast have effects on the absolute values of RGB, so that the fluorescence images of detected samples should be recorded along with the standard samples to improve the accuracy. The blank background and PDMS setup without any samples shows a very low red intensity at ~4.6 and ~7.08, probably originating from the excitation light source and light scattering.

References

[1] U. Rambabu, D. Amalnerkar, B.K. Buddhudu, Fluorescence spectra of Eu^{3+}-doped $LnVO_4$ (Ln = La and Y) powder phosphors. Mater. Res. Bull **35**, 929-936 (2000). https://doi.org/10.1016/S0025-5408(00)00287-7

[2] X. Zheng, C. Wang, J. Dai, W. Shi, Y. Yan, Design of mesoporous silica hybrid materials as sorbents for the selective recovery of rare earth metals. J. Mater. Chem. A **3**, 10327-10335 (2015). https://doi.org/10.1039/C4TA06860B
